# Acute ischaemic stroke: recent advances in reperfusion treatment

**DOI:** 10.1093/eurheartj/ehac684

**Published:** 2022-12-07

**Authors:** Petr Widimsky, Kenneth Snyder, Jakub Sulzenko, Leo Nelson Hopkins, Ivana Stetkarova

**Affiliations:** Cardiocenter, Charles University and University Hospital Kralovske Vinohrady, Ruska 87, Prague 10, Czech Republic; Department of Neurosurgery, Jacobs School of Medicine and Biomedical Sciences, University at Buffalo, Buffalo, NY, USA; Cardiocenter, Charles University and University Hospital Kralovske Vinohrady, Ruska 87, Prague 10, Czech Republic; Department of Neurosurgery, Jacobs School of Medicine and Biomedical Sciences, University at Buffalo, Buffalo, NY, USA; Department of Neurology at the Third Faculty of Medicine, Charles University and University Hospital Kralovske Vinohrady, Ruska 87, Prague 10, Czech Republic

**Keywords:** Acute stroke, Endovascular, Thrombectomy, Thrombolysis

## Abstract

During the last 5–7 years, tremendous progress was achieved in the reperfusion treatment of acute ischaemic stroke during its first few hours from symptom onset. This review summarizes the latest evidence from randomized clinical trials and prospective registries with a focus on endovascular treatment using stent retrievers, aspiration catheters, thrombolytics, and (in selected patients) carotid stenting. Novel approaches in prehospital (mobile interventional stroke teams) and early hospital (direct transfer to angiography) management are described, and future perspectives (‘all-in-one’ laboratories with angiography and computed tomography integrated) are discussed. There is reasonable chance for patients with moderate-to-severe acute ischaemic stroke to survive without permanent sequelae when the large-vessel occlusion is removed by means of modern pharmaco-mechanic approach. Catheter thrombectomy is now the golden standard of acute stroke treatment. The role of cardiologists in stroke is expanding from diagnostic help (to reveal the cause of stroke) to acute therapy in those regions where such up-to-date Class I. A treatment is not yet available.

## Graphical Abstract

**Graphical Abstract:**
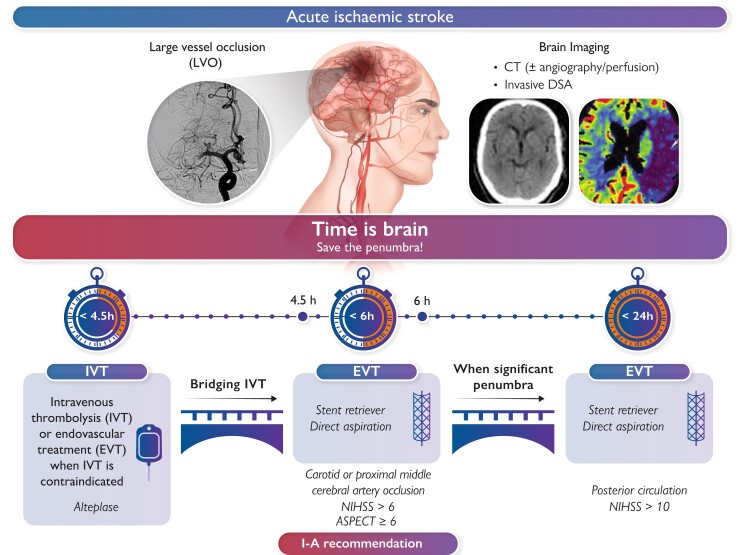
Upper part left: angiography of an occluded middle cerebral artery (typical image in an acute anterior circulation stroke). Upper middle: ongoing ischaemia in the brain area, supplied by the occluded artery. Upper right: computed tomography and perfusion computed tomography images in the acute phase of anterior circulation stroke. Lower part left: intravenous thrombolysis is recommended within 4.5 h of symptom onset (if no contraindication is present). Lower middle: endovascular treatment (i.e. thrombectomy with stent retriever and/or aspiration catheter) is recommended within 6 h of symptom onset, irrespective of whether intravenous thrombolysis was given or not. Lower right: Time window for endovascular treatment may be extended up to 24 h from symptom onset when significant penumbra is proven by advanced imaging methods (e.g. perfusion computed tomography). ASPECT, Alberta Stroke Programme Early CT; CT, computed tomography; DSA, digital subtraction angiography; EVT, endovascular treatment; IVT, intravenous thrombolysis; LVO, large-vessel occlusion; NIHSS, National Institutes of Health Stroke Scale.

## The importance of interdisciplinary approach to stroke

Acute ischaemic stroke incidence rates are similar to those of acute coronary syndromes. Both these acute ischaemic syndromes are responsible for vast majority of death from cardiovascular diseases and thus for total mortality in most countries. Acute stroke is probably the most important acute serious illness, where interdisciplinary approach is essential to offer the best chance for survival and functional recovery of patients. Traditionally, the closest cooperation was between neurologists, neurosurgeons, and radiologists,^1^ and the implementation of an interdisciplinary ‘stroke code’ protocol may shorten the in-hospital delays by almost 30 min.^2^

While cooperation between these specialists is essential during the acute hospital admission, the involvement of cardiologists was traditionally more focused on stroke prevention as cardiovascular diseases (especially hypertension and atrial fibrillation) are among the most frequent causes of ischaemic stroke. The growing evidence supporting the role of stroke thrombectomy triggered interest among interventional cardiologists to help with the widespread implementation of thrombectomy into stroke services. The European Society of Cardiology Council on Stroke published two position papers,^3,4^ defining several roles cardiologists should have in stroke—including thrombectomy in regions, where this treatment is not available. Interventional cardiologists, who plan to join the interdisciplinary stroke teams, should undergo a formal training in stroke thrombectomy^5^ with full respect to the leading role of neurologists in acute stroke treatment. In those (still only few) hospitals, where acute ischaemic stroke thrombectomy is performed by interventional cardiologists, the decision for thrombectomy as well as post-procedural therapy is led by neurologists. Such interdisciplinary cooperation offers a better chance for survival without serious disability for acute stroke patients.

The aim of this review paper is to summarize the current knowledge on acute ischaemic stroke diagnosis and treatment for the clinical cardiologists and other specialists involved in stroke care and to show areas, where better interdisciplinary cooperation may further improve the outcomes of stroke patients (*Graphical Abstract*).

## Prehospital management, transport delays

The phrase ‘time is muscle’ is used by cardiologists to simply describe the importance of time during the acute phase of myocardial infarction in order to save the myocardium in jeopardy. Similarly, ‘time is brain’ is used by neurologists to save penumbra (ischaemic, but still viable brain tissue in the acute phase of ischaemic stroke). While primary percutaneous coronary intervention (pPCI) in ST-elevation myocardial infarction (STEMI) is typically indicated up to 12 h from symptom onset, a similar time window for thrombectomy in acute ischaemic stroke is 6 h. This can be extended up to 24 h if patients meet the DAWN trial criteria.^6^ The critical role of time delays in acute ischaemic stroke was described by the HERMES collaborators.^7^ In this meta-analysis of patients with large-vessel occlusion (LVO) ischaemic stroke, the earlier treatment with endovascular thrombectomy was associated with lower degrees of disability at 3 months. Benefit became nonsignificant with total ischaemic time >7.3 h.

What are the individual components of the treatment delay? The median total prehospital time for emergency medical services (EMSs) ground transports in the USA in 2018–19 was 35 min [interquartile range (IQR): 27–45, 90th percentile 58], while for air transports, it was substantially longer at 56 min (IQR: 43–70, 90th percentile 86).^8^ The door-in-door-out (DIDO) time is an important metric for stroke centres without an on-site mechanical thrombectomy service. The median DIDO time was 67 min (IQR: 55–94) in a recent single-centre study.^9^ Another study described the following median time intervals: DIDO 85 min for the primary stroke centre, interfacility transport door-out-door-in (DODI) 26 min, comprehensive stroke centre door-to-arterial puncture 21 min, and puncture to recanalization 24 min.^10^

**Figure 1:**
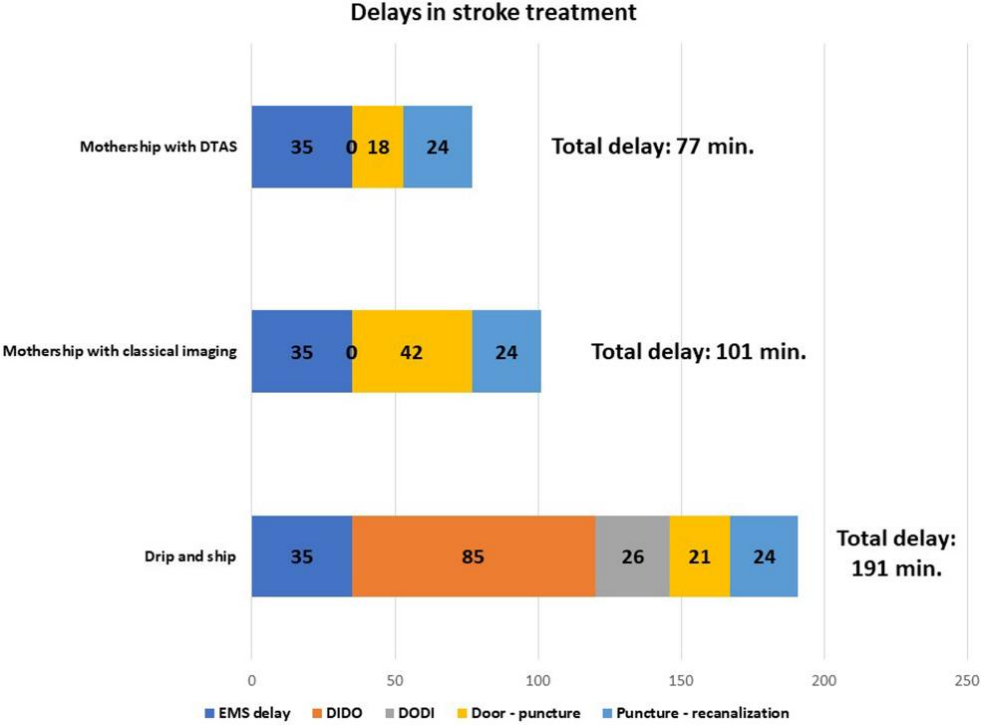
Drip-and-ship (presentation to non-thrombectomy centre followed by interhospital transfer to thrombectomy centre): emergency medical service delay (call-to hospital): 35 min. Door-in-door-out in the primary hospital: 85 min. Secondary transport (DODI): 26 min. Door-to-arterial puncture: 21 min. Puncture to recanalization: 24 min. Mothership with classical imaging strategy (admission to a thrombectomy centre with noninvasive imaging first): emergency medical service delay (call-to hospital): 35 min. Door-to-arterial puncture: 42 min. Arterial puncture to recanalization: 24 min. Mothership with direct transfer to angiography suite (admission to a thrombectomy centre with direct transfer to angiography suite, i.e. ST-elevation myocardial infarction -like strategy): emergency medical service delay (call-to hospital): 35 min. Door-to-arterial puncture: 18 min. Arterial puncture to recanalization: 24 min. Total ‘call-to recanalization’ time: 77 min.

*Figure 1* summarizes these time delays for different strategies.^8–10^ It can be hypothesized that a long-distance mothership model may be beneficial for the patients for up to 85 min^10^ or 90 min (difference of 191–101 min) of EMS time. This is feasible almost everywhere in developed countries, and thus the strategy for acute stroke should follow the STEMI strategy: patients with pre-defined criteria should be transferred almost exclusively to the thrombectomy centres and not to local stroke centres without thrombectomy facilities/teams. The alternative might be the Mobile Interventional Stroke Team (MIST).

The NYC MIST trial demonstrated that the use of a MIST travelling to perform endovascular thrombectomy in a hospital where the patient is first diagnosed was faster and led to improved discharge outcomes, when compared with the drip-and-ship (DS) model. Especially, the patients presenting within ≤6 h from stroke onset had better clinical outcomes in the MIST model (54% had a good 90-day outcome), when compared with 28% in the DS model.^11^ The MIST model may be optimal for centres with good technical equipment, but insufficient staff to cover 24/7 thrombectomy service. When the local thrombectomy team is not available 24/7 in a stroke centre (due to the lack of neuroradiologists), this gap may be overcome with the help of local interventional cardiologists involved in thrombectomy services.^12^

The shortest in-hospital delays may be achieved with direct transfer to angiography suite (DTAS). DTAS has shown encouraging results in reducing in-hospital delays. DTAS allows bypassing of conventional imaging by using flat-panel computed tomography (FP-CT; sometimes also called low contrast imaging). Ideal DTAS candidates are patients admitted in the early window with severe symptoms. Some centres use FP-CT before femoral puncture, and others prefer the additional time savings by directly assessing the presence of LVO with an angiogram. The latter, however, leads to unnecessary arterial punctures in patients with no LVO (3%–22% depending on selection criteria). DTAS has been shown to be effective and safe in improving in-hospital workflow, achieving a reduction of door-to-puncture time as low as 16 min without safety concerns.^13^ The future certainly is a combined laboratory, where an angiograph and a 320 slice CT are combined ‘all-in-one’.

In a recent study from Barcelona, the DTAS protocol decreased the median door-to-arterial puncture time [18 min (IQR: 15–24 min) vs. 42 min (IQR: 35–51 min); *P* < 0.001] and door-to-reperfusion time [57 min (IQR: 43–77 min) vs. 84 min (IQR: 63–117 min); *P* < 0.001]. The DTAS protocol decreased the severity of disability across the range of the modified Rankin scale (mRs) [adjusted common odds ratio (OR): 2.2; 95% confidence interval (CI): 1.2–4.1; *P* = 0.009]. Safety variables were comparable between groups.^14^

**Figure 2:**
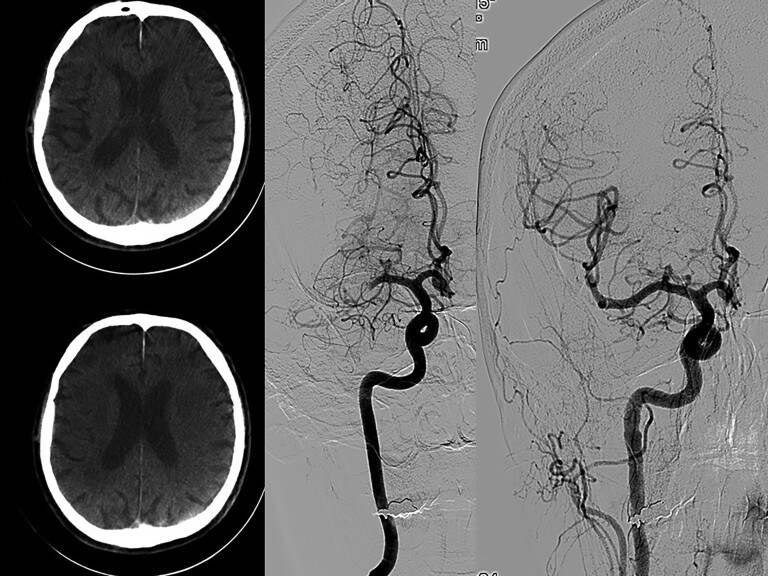
Flat detector computed tomography images (left) with no signs of intracranial bleeding and no clear signs of developed ischaemia. Selective angiogram done immediately after flat detector computed tomography with the same equipment showing middle cerebral artery occlusion (middle) and reopening after thrombectomy (right).

*Figure 2* shows flat detector CT images with no signs of intracranial bleeding and no clear signs of developed ischaemia. A selective angiogram is done immediately after flat detector computed tomography with the same equipment showing middle cerebral artery occlusion and reopening after thrombectomy.

## Early in-hospital stroke diagnosis

**Figure 3:**
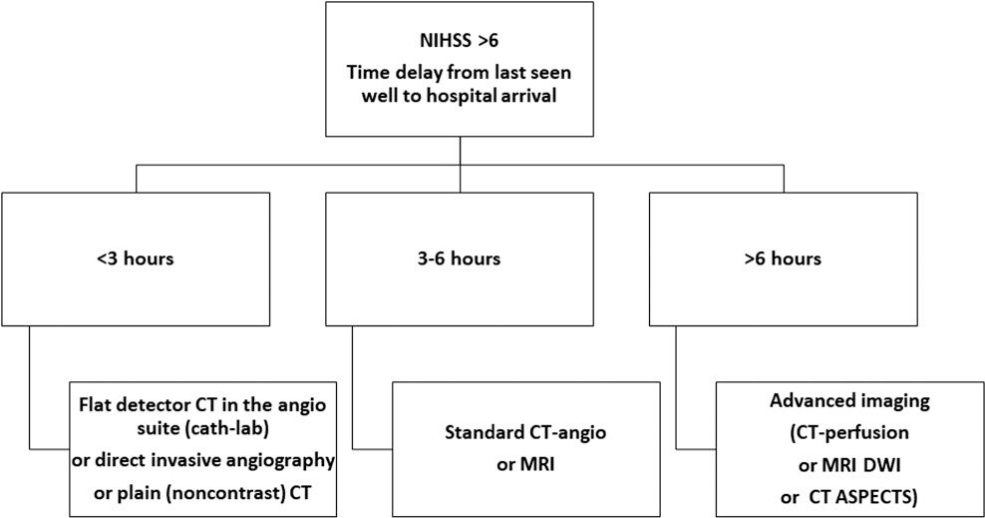
Possible modes of early diagnostic approach to acute stroke to select patients for acute thrombectomy.

The options for the diagnostic approach in the first minutes after arrival to a stroke centre are summarized in *Figure 3*. This approach applies only to moderate or severe strokes [high on the National Institutes of Health Stroke Scale (NIHSS)], because of the high likelihood of LVO, amenable to thrombectomy.

The reason why patients presenting after 6 h should undergo advanced imaging is that in many of these patients, the ischaemic core (irreversible brain damage) is already too large and penumbra (reversible ischaemia) is only small. Opening the occluded artery, which is supplying irreversibly damaged brain tissue, cannot improve the outcome, but substantially increases the risk of haemorrhagic transformation and symptomatic intracerebral bleeding. Nevertheless, a significant proportion of these late presenters may still benefit from thrombectomy due to the existing collateral flow or due to intermittent or incomplete obstruction. The meta-analysis of individual data of 505 stroke patients with evidence of reversible cerebral ischaemia treated between 6 and 24 h after last seen well (AURORA collaboration group^15^) found benefit of endovascular thrombectomy. These findings suggest that in such patients, thrombectomy should not be withheld on the basis of the mode of presentation or of the point in time of presentation within the 6–24 h time window. In fact, the data from the DAWN and DEFUSE 3 trials^16^ led to the guidelines update, suggesting thrombectomy suitability up to 24 h in patients who meet the entry criteria of these two trials.

## The role of thrombolysis in the current era of stroke thrombectomy

The first (small) randomized trial showing potential benefits of intravenous thrombolysis (IVT; when used early in the course of acute ischaemic stroke) was published in 1992.^17^ The first positive randomized trial of thrombolysis was published in 1995.^18^ This trial proved improvement in neurologic functional outcomes (43% after thrombolysis and 27% after placebo), but the overall mortality was not significantly different (17.3% after thrombolysis vs. 20.5% after placebo, *P* = 0.30). Symptomatic intracranial (6.4% thrombolysis vs. 0.6% placebo) as well as overall fatal (2.9% thrombolysis vs. 0.3% placebo) bleeding was significantly higher after recombinant tissue plasminogen activator (rt-PA).

An important meta-analysis^19^ included 3670 patients from 8 trials using i.v. rt-PA and was focused on the time window between symptom onset and start of thrombolysis. Favourable 3-month outcome (defined as mRs 0–1) increased as time delay decreased (*P* = 0.0269) and there was no benefit of rt-PA treatment beyond 270 min. The benefit was greater, the earlier patients were treated: adjusted odds of a favourable 3-month outcome were 2.55 (95% CI: 1.44–4.52) for 0–90 min, 1.64 (95% CI: 1.12–2.40) for 91–180 min, 1.34 (95% CI: 1.06–1.68) for 181–270 min, and a nonsignificant 1.22 (95% CI: 0.92–1.61) for 271–360 min. Large intracerebral haemorrhage occurred in 5.2% of patients assigned to alteplase and 1.0% of controls, with no relationship to time delays. Mortality increased with time delay: only thrombolysis given within 90 min of stroke onset decreased mortality, while thrombolysis used after more than 270 min increased mortality.

A recent Australian trial^20^ investigated, whether IVT used between 4.5 and 9 h after stroke onset in patients having hypoperfused but salvageable brain regions detected on perfusion imaging could improve the outcomes compared with placebo. Significantly, more patients (35.4% vs. 29.5%, *P* = 0.04) in the thrombolysis group had good neurologic outcomes, but this came at the price of higher rates of symptomatic intracerebral haemorrhage (sICH; 6.2% vs. 0.9%, *P* = 0.053) and numerically (not significantly) higher all-cause mortality at 3 months (11.5% vs. 8.9%, *P* = 0.67).

The question of whether the combination of delayed (4.5–9 h after stroke onset) IVT and mechanical thrombectomy may be deleterious (e.g. increased risk of intracranial haemorrhage or clot migration to more distal vessel segments not accessible for thrombectomy) was tested in two small studies with neutral results.^21,22^

Intra-arterial thrombolysis alone was compared with a placebo in the PROACT-II trial.^23^ Intra-arterial thrombolysis achieved better neurologic outcomes, but there was no difference in mortality. Intra-arterial thrombolysis increased the risk of sICH more than five times (11% vs. 2% in the placebo group).

Combining intravenous and intra-arterial alteplase was investigated and frequently used until 2004, when intra-arterial alteplase was quickly replaced by mechanical thrombectomy, which demonstrated more frequent and more complete recanalization of LVO.^24^

Intra-arterial thrombolysis after successful thrombectomy was tested in a small, prematurely terminated trial^25^: The 121 patients treated with thrombectomy within 24 h after stroke onset were randomized to intra-arterial alteplase (maximum dose 22.5 mg) infused over 15–30 min or placebo. Excellent neurologic outcome (mRs of 0 or 1 at 90 days) was 59% with alteplase and 40% with placebo (adjusted risk difference: 18.4%; 95% CI: 0.3%–36.4%; *P* = 0.047). The 90-day mortality was 8% with alteplase and 15% with placebo (risk difference: −7.2%; 95% CI: −19.2% to 4.8%).

There are very few data about the use of intra-arterial thrombolysis after failed thrombectomy—the authors of this manuscript are rather sceptical in this respect and are not using thrombolysis in situations when thrombectomy failed to achieve successful reperfusion—with one possible exception: when the thrombotic occlusion site is too distal and not safely accessible with a stent retriever. One additional area of exploration is combined intra-arterial thrombolysis and thrombectomy when a residual distal clot is present after LVO is removed and there is a TICI 2a or less result on the final angiogram.

The DIRECT-MT trial compared intravenous alteplase before endovascular treatment (EVT) vs. EVT alone in 656 patients. Intravenous alteplase was more frequently (19% vs. 10%) associated with early reperfusion. The effect was modified by time from randomization to groin puncture: thrombolysis was superior only when this time delay exceeded 33 min. Thus, intravenous alteplase should be considered before thrombectomy if a groin puncture delay of more than 30 min is anticipated by the treating medical team.^26^

The MR CLEAN-NO IV trial enrolled 539 stroke patients eligible for thrombolysis and thrombectomy who presented to a thrombectomy-capable hospital. Patients were randomly assigned to receive direct thrombectomy or IVT followed by thrombectomy. The primary endpoint (good functional outcome defined as mRs 0–2 at 90 days) was present in 49% (direct thrombectomy) and 51% (thrombolysis + thrombectomy). Mortality was 20.5% with thrombectomy alone and 15.8% with thrombolysis plus thrombectomy (adjusted OR: 1.39; 95% CI: 0.84–2.30). Symptomatic intracerebral haemorrhage occurred in 5.9% and 5.3% of the patients in the respective groups (adjusted OR: 1.30; 95% CI: 0.60–2.81). The authors concluded that direct thrombectomy was neither superior nor non-inferior to IVT followed by thrombectomy.^27^

Similarly, the third study comparing direct thrombectomy with bridging thrombolysis followed by thrombectomy (the SKIP trial) did not find a significant difference between both strategies.^28^

Promising results with the combination of intravenous tenecteplase (TNK) and mechanical thrombectomy were obtained by the EXTEND-IA TNK Investigators. Intravenous TNK achieved more frequent angiographic reperfusion and tended to achieve better clinical outcomes in several parameters without increasing risk of bleeding.^29^

## Recent advances in endovascular treatment

### The key milestone trials

EVT of acute ischaemic stroke showed clear benefit in selected group of patients with LVO. Several randomized trials published after 2015^30–35^ demonstrated better clinical outcomes in patients who underwent EVT for carotid or proximal middle cerebral artery occlusion, within 6 h after symptom onset with significant neurological deficit (NIHSS >6) and small early ischaemic changes on CT [Alberta Stroke Programme Early CT Score (ASPECTS) ≥6] compared with best medical treatment including IVT. These ground-breaking trials led to changes in guidelines and EVT is now IA recommendation in this selected group of patients with acute ischaemic stroke.

### Patients with late presentation

Another milestone that extended the time window for EVT was the publication of DEFUSE 3 a DAWN trials.^6,36^ These trials demonstrated the benefit of EVT in patients with carotid or proximal middle cerebral artery occlusion presenting 6–16 or 6–24 h after symptom onset, respectively. Patients were selected for treatment based on ischaemic core-penumbra mismatch evaluated on CT perfusion or diffusion-weighted magnetic resonance imaging (DWI-MRI).

Recently, several trials were published looking to expand the population of patients who would benefit from endovascular stroke treatment, but were excluded from previous randomized trials.

### Large ischaemic core strokes

Previous trials mostly excluded patients with an ASPECTS <6, thus the benefit of EVT in patients with large ischaemic core strokes was unknown. Several recently published or ongoing trials are addressing this issue.

A prespecified secondary analysis of the SELECT study included 105 patients with ASPECTS ≤5 or ischaemic cores of ≥50 cm^3^ on CT perfusion. Thirty-one per cent of patients in the EVT group achieved independence at 90 days vs. 14% in the medical group (OR: 3.27; 95% CI: 1.11–9.62; *P* = 0.03).^37^ The signal of benefit was especially promising for patients with ASPECTS 3–5 and a core <100 cm^3^. The SELECT 2 (NCT number: NCT03876457) is currently recruiting patients with these specific inclusion criteria.

A recently published Japanese trial randomized patients with ASPECTS 3–5 within 6 h after they were last seen well or within 24 h if there was no early change on fluid-attenuated inversion recovery images. The results showed better clinical outcomes defined as mRs of 0–3 at 90 days in 31.0% of patients in the endovascular-therapy group compared with 12.7% in the medical-care group (relative risk: 2.43; 95% CI: 1.35–4.37; *P* = 0.002). Higher rates of intracranial bleeding were observed in the endovascular group (58.0% vs. 31.4%, *P* < 0.001).^38^ Some criticism emerged firstly in shifting the definition of good clinical outcome to mRs 0–3 and also that the dosage of IVT used in this trial was lower than usual which could reduce the effect in medical-care group. Possible limitations of different methods evaluating large ischaemic core size in acute stroke were recently discussed in a commentary,^39^ appropriately stressing the practical advantage of CT-ASPECTS over DWI-MRI ASPECTS.

Other ongoing studies are investigating EVT for patients with large ischaemic cores and different time settings: TESLA (ASPECTS 2–5 within 24 h of last known normal, NCT03805308), TENSION (ASPECTS 3–5 within 12 h, NCT03094715), and LASTE (ASPECTS 0–5 within 7 h, NCT03811769).

### Mild strokes with proximal occlusion

On the opposite site of the spectrum stands the cases where the patient has proximal LVO but only a mild (NIHSS 0–5) neurological deficit. Previous randomized trials did not generally include these patients, so the benefit of EVT is unknown.

Two large observational studies found no difference between EVT and the best medical therapy for these patients.^40,41^ In both trials, EVT was associated with higher odds of sICH, though this was not reflected in the overall outcome.

These results show that EVT may not be the best option for all patients with LVO and mild stroke. On the other hand, the rescue strategy of performing thrombectomy after neurologic deterioration seems to be associated with worse outcomes than immediate EVT.^42^ Subgroup analyses of previously mentioned trials are suggesting that patients who could benefit from EVT despite mild neurologic deficit at the presentation are those with more proximal, larger clots who are not candidates for IVT. EXTREMIS-MOSTE, Tempo-2 (NCT02398656), and ENDO-LOW (NCT04167527) trials are testing a similar hypothesis in patients with mild strokes and proximal LVO.

### Devices and techniques

In the first crucial randomized trials,^30–35^ the vast majority of cases were performed with second-generation stent retrievers. The use of balloon guide catheters became the standard of care when using stent retrievers after several trials proved the benefits of achieving faster recanalization, a more often first-pass effect, and improved clinical outcomes compared with proximal large bore conventional guide catheters.^43,44^

The choice between stent retriever and direct aspiration is still primarily driven by institutional and operator preferences. Three randomized controlled trials demonstrated the noninferiority of contact aspiration to stent retriever thrombectomy with similar procedural and clinical performance between the two techniques.^45–47^ Suggestions now occurred that each technique could be more effective in different specific settings. Stent retrievers seem to perform better when the thrombus is soft, red blood cell rich (which is represented by the hyperdense vessel sign on initial non-contrast CT or the blooming/susceptibility sign on magnetic resonance imaging). On the contrary, direct aspiration seems to perform better in cases of fibrin rich thrombus, when these imaging signs are not present.^48,49^

The technology is moving fast, new designs of stent retrievers with new functions like radial force adaptation are emerging. The same applies to aspiration catheters. Larger inner diameters and more flexible devices are available. The question of whether the technological improvements will have an impact on clinical outcomes remains to be answered in randomized trials.

## Endovascular treatment in specific locations

### Posterior circulation

**Figure 4:**
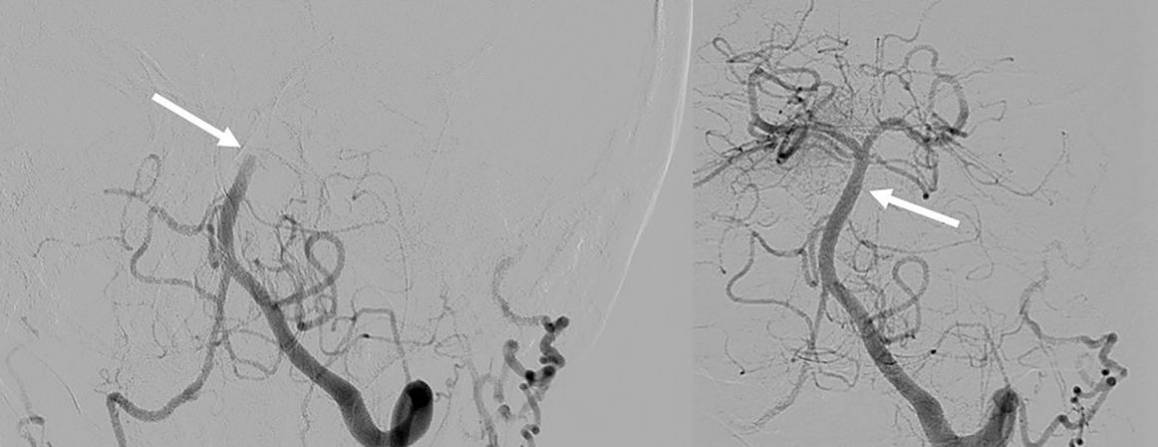
Typical case of basilar artery occlusion (left side). TICI 3 recanalization achieved using direct aspiration technique (right side).

A typical example of posterior circulation stroke [basilar artery occlusion (BAO)] treated with thrombectomy is given in *Figure 4*.

Two randomized trials evaluating the efficacy and safety of EVT in patients with BAO were recently published.

The BASICS trial^50^ randomized 300 patients with BAO presenting within 6 h of stroke onset to either EVT or medical therapy alone, including IVT in ∼80% of the patients. The trial showed that EVT did not provide superior clinical outcomes over standard therapy for this group of patients (90-day mRs 0–3: 44.2% in EVT vs. 37.7% in controls; adjusted OR: 1.18; 95% CI: 0.92–1.50). However, subgroup analysis suggested a benefit in EVT patients with a baseline NIHSS score ≥10 with significantly higher changes of favourable outcomes at 90 days (mRs score 0–3 risk ratio: 1.45; 95% CI: 1.03–2.04) than the control group. The 90-day mortality was 38.3% in the EVT and 43.2% in the control group, and the rates of sICH were 4.5% in the EVT and 0.7% in the control group.

The BEST trial also compared EVT to the best medical management in patients with BAO presenting within 8 h of stroke onset.^51^ The study was terminated early because of a high crossover rate (13% of rescue EVT when patients deteriorated neurologically) and poor recruitment. In the intention-to-treat analysis, there was no evidence of a difference in the proportion of participants with 90-day mRs 0–3 at 90 days: 42% of 66 patients in the intervention group vs. 32% of 65 in the control group (adjusted OR: 1.74; 95% CI: 0.81–3.74).

Two BAO trials recently presented at the European Stroke Organization Congress 2022 demonstrated the benefit of EVT (in terms of the functional outcome mRs 0–3) compared with the best medical therapy (the ATTENTION trial^52^ and the BAOCHE trial—NCT02737189).

The emerging evidence of EVT benefit in patients with BAO justifies the clinical practice of many centres to offer EVT routinely within 24 h from last known well as part of a life-saving rescue strategy due to the devastating neurological nature of this life-threatening clinical situation.

### Tandem lesions and the role of acute carotid stenting

**Figure 5:**
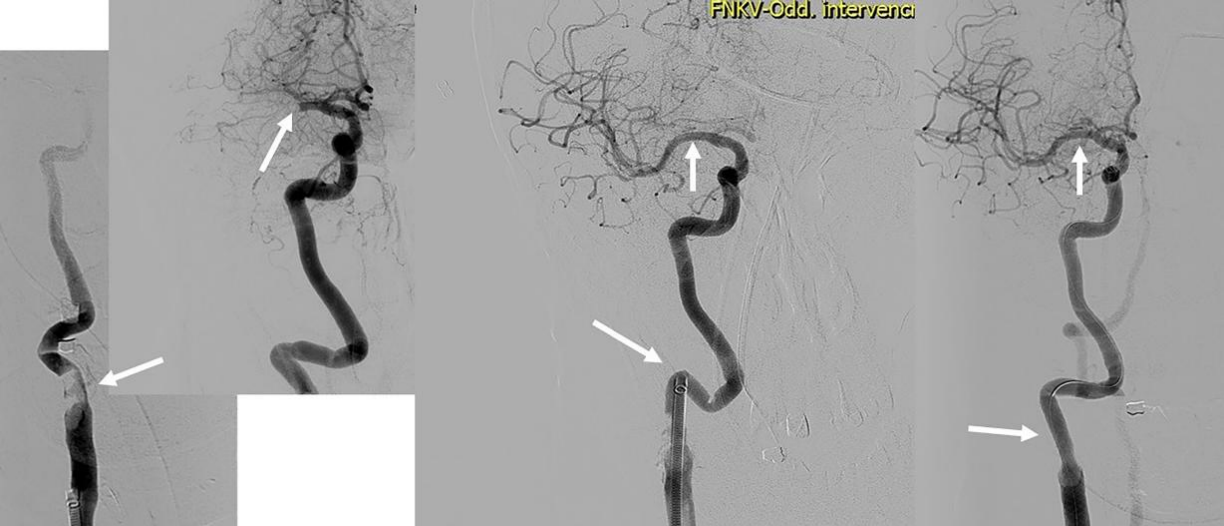
Tandem lesion. Angiogram showing ruptured atherosclerotic plaque with large thrombus in the carotid bifurcation causing the occlusion of external carotid artery and near occlusion of proximal internal carotid artery (first left). Selective intracranial angiogram acquired after passing through the proximal internal carotid artery near occlusion is showing proximal middle cerebral artery occlusion (middle left). Intracranial recanalization was achieved first, using direct aspiration technique with a large bore sheath placed behind the proximal internal carotid artery lesion (middle right). Final result after implantation of the stent to proximal internal carotid artery (right).

The situations where the extracranial internal carotid artery or vertebral artery is severely stenosed or occluded in association with the middle cerebral artery or BAO are usually more technically challenging and time consuming. The effect of EVT in acute strokes caused by the tandem lesion in anterior circulation (see example in *Figure 5*) was comparable with the non-tandem occlusion in the HERMES trial.^35^ The situation nevertheless carries several considerations that are being evaluated.

The first consideration regards the technical approach. Retrograde approach means to recanalize intracerebral occlusion first and to treat the extracranial lesion second. In antegrade approach, the extracranial lesion is treated as the first one. Most of the studies found no difference between these two approaches regarding clinical outcomes, rates of successful recanalization, or times from puncture to recanalization. Some non-randomized retrospective studies, however, showed better clinical outcomes in the retrograde approach^53^ as well as faster time to recanalization in the retrograde approach.^54^

Endovascular management of tandem occlusions is usually complex with the potential need of acute stenting of the extracranial carotid artery steno-occlusive lesion along with the additional need of antithrombotic therapy initiation and thus increasing the risk of intracerebral haemorrhage. The concerns about a high rate of sICH when combining IVT with an early antiplatelet therapy were based on the ARTIS trial conducted in the pre-EVT era.^55^ Newer data from EVT studies are nevertheless suggesting that the use of acute stenting of the extracranial carotid lesion (with the need to administer early antiplatelet therapy) was associated with better reperfusion without an excess risk of sICH or mortality.^56–58^ Most centres use now immediate (acute phase) carotid stenting approach rather than deferred stenting. However, some data demonstrated a 16% risk of intracranial bleeding when carotid stenting is performed during the acute phase of stroke, due to the need for more potent antithrombotic therapy after stent implantation.^59^ The impact on clinical outcome of such approach is now being evaluated in the randomized trial TITAN (NCT03978988).

### Distal medium-vessel occlusion

Meta-analyses of HERMES collaboration data from patients with proximal, large-diameter M2 segment middle cerebral artery occlusions showed benefit from EVT in such cases (90-day mRs 0–2 58.2% for EVT vs. 39.7% for medical management; adjusted OR: 2.39, 95% CI: 1.08–5.28, *P* = 0.03).^60^ With recent advances in thrombectomy technology, distal, medium-vessel occlusions (DMVOs) are now emerging as a promising next potential EVT frontier.

Randomized data for DMVOs are lacking, and there are just few case series reporting outcomes in these cases. Grossberg *et al.*^61^ performed thrombectomy in 69 patients with anterior cerebral artery (43%), M3 middle cerebral artery (54%), or posterior cerebral artery (10%) occlusions, including 62% primary distal occlusions and 33% distal occlusions present due to distal embolization during EVT. Outcomes included substantial reperfusion (mTICI 2b−3) in 83%, parenchymal haematoma in the distal arterial field in 4%, 90-day functional independence in 30%, and mortality in 20%. While similar data are suggesting angiographic success and safety of EVT for DMVOs, randomized controlled trials are needed to establish any benefit of EVT for more distal occlusions.

## Antithrombotic medication during thrombectomy

While unfractionated heparin (or other parenteral anticoagulants, e.g. low molecular weight heparins) plays a key role in antithrombotic treatment of acute coronary syndromes, they are contraindicated (at least in full therapeutic doses) in acute ischaemic stroke. There are several reasons for contraindication of full therapeutic anticoagulant use in stroke: haemorrhagic stroke (15% of all strokes, frequently difficult to differentiate based only on clinical signs), risk of haemorrhagic transformation of ischaemic stroke (especially large ischaemic strokes, i.e. those suitable for thrombectomy are at risk), uncontrolled hypertension, pre-existing anticoagulant therapy, etc.

A systematic review,^62^ a total of 24 trials involving 23 748 patients, examined the effect of anticoagulant therapy vs. control in the early treatment of acute ischaemic stroke. The major findings are: (i) anticoagulant therapy did not improve all-cause mortality, (ii) anticoagulants did not reduce disability (dependence), (iii) anticoagulant therapy was associated with about 9 fewer recurrent ischaemic strokes per 1000 patients treated, but it was also associated with a 9 per 1000 increase in sICH, (iv) anticoagulants avoided about 4 pulmonary emboli per 1000, but this benefit was offset by an extra 9 major extracranial haemorrhages per 1000.

The data on periprocedural heparin during mechanical thrombectomy are surprisingly sparse. A Chinese retrospective registry^63^ demonstrated that heparinization during thrombectomy did not significantly influence recanalization and mortality rates, but increased sICH rates and distal embolization risk. Furthermore, heparinization during thrombectomy was an independent predictor for poor outcome (OR: 1.79, 95% CI: 1.23–2.59, *P* < 0.01). The periprocedural heparin doses in this registry varied between 50 and 100 IU/kg of body weight. It seems likely, that low-dose heparin (e.g. 20–30 U/kg) may offer better results. A randomized multicentre trial testing the effect of periprocedural medication: acetylsalicylic acid, unfractionated heparin, both, or neither^64^ is underway and may respond several questions on periprocedural medication.

The standard practice in most neuro interventional centres is based on the use of IVT before thrombectomy (thrombolysis-facilitated mechanical thrombectomy or bridging thrombolysis). When a stroke patient enters angio-suite with running infusion of tPA, usually no anticoagulation (or a minimal heparin dose just to protect against catheter thrombi) is used. In patients who are not treated with thrombolysis (presentation after >270 min from stroke onset or contraindication to tPA), usually a low dose of heparin (10–30 IU/kg) is used during mechanical intervention.

## Periprocedural management

Endovascular procedures for acute ischaemic stroke may require more staff in the angio-room compared with pPCI for STEMI. The optimal scenario includes six healthcare professionals: the first operator (neurointerventional specialist), the second operator (assisting young physician in training or cath-lab nurse), an intensivist (to take care of the clinical status of the patient, manage blood pressure, intubate if necessary, etc.), a neurologist, one nurse, and one radiology assistant. The minimum staff during a thrombectomy procedure is four healthcare professionals (on the contrary, the minimal staff for pPCI in STEMI may be just one cardiologist and one nurse).

The role of intubation and general anaesthesia (vs. simple conscious sedation) during mechanical thrombectomy remains unclear. One study randomized 128 patients to general anaesthesia vs. conscious sedation. Successful reperfusion was higher and clinical outcomes were better in general anaesthesia.^65^ A more recent and larger (2013 patients) study^66^ found the opposite: general anaesthesia during thrombectomy was associated with worse 3-month functional outcomes. When the patient is awake, the brain is autoregulating. The anaesthesiologist often drops the blood pressure into a range that further reduces perfusion to the penumbra, and thus ischaemia progression is faster. Indeed, the risk of general anaesthesia is associated with large variations in blood pressure during intubation and in time delays related to intubation and ventilatory support arrangements. The risk of conscious sedation is patient unrest and movement. Most centres decide individually—use conscious sedation as the default strategy and intubate the patient whenever indicated.

It is not known what the optimal periprocedural blood pressure is. One study showed that higher systolic blood pressure was associated with less functional independence at 3 months in patients with successful recanalization and with more sICH regardless of recanalization status.^67^ However, a recent comprehensive review underlines the importance of an individualized approach to blood pressure management during thrombectomy.^68^ In fact, practical experience showed that watching blood pressure during thrombectomy is a sign of success: the operator sees the normalization of previously higher blood pressure quickly after clot removal. This may be a physiologically ideal scenario: higher blood pressure when the artery is still occluded enables better collateral flow and, thus, penumbra preservation and pressure normalization shortly after clot removal decrease the risk of haemorrhagic transformation.

## Periprocedural complications related to thrombectomy

### Clinically recognized complications

In general, two complications have major clinical relevance: sICH and stroke extension due to embolization of another vascular territory. The risk of sICH after mechanical thrombectomy does not differ from sICH rates after IVT or from sICH risk when both treatment modalities are used together (bridging thrombolysis)—rates vary in different trials in the range of 2%–9%.^31,32,69^ The major risk factor for sICH development (besides changes in blood coagulation due to thrombolytics) is late reperfusion—if the vessel is reopened at the time when large ischaemic core dominates over penumbra, ICH is more likely. Clinically detected stroke extension caused by clot fragment embolization to another vascular territory (e.g. clot retrieved from middle cerebral artery and fragment embolize to anterior cerebral artery) is rather rare (1%–3%). Somewhat higher rates (up to 5%) are observed angiographically without clinical stroke progression.

### Imaging-detected complications

CT or magnetic resonance detects asymptomatic haemorrhagic transformation (any ICH) in about 10%–13% of patients, i.e. ∼3–4 times more frequently than clinically observed sICH. Also this asymptomatic ICH occurs with the same rates after IVT as after mechanical thrombectomy. Very rare are gross mechanical complications caused by catheter manipulation: arterial dissection, rupture, perforation, or arteriovenous fistula. They are usually clinically relevant and are covered under sICH.

### Access-site complications

Access-site complications (groin haematoma, arteriovenous femoral fistula) occur similarly to any other invasive endovascular procedures, i.e. clinically relevant in 1%–2%.

### Complications related to general anaesthesia

Complications related to general anaesthesia do not differ from such complications in any general anaesthesia. Special importance in stroke has blood pressure drop during intubation (may greatly facilitate ischaemic core progression) and time delays related to intubation and insertion of artificial ventilation (also causes stroke progression).

## Conclusions

The diagnosis and treatment of acute ischaemic stroke changed dramatically during last 5–7 years. There is reasonable chance for patients with moderate-to-severe acute ischaemic stroke to survive without permanent sequelae when the LVO is removed by means of modern pharmaco-mechanic approach. Catheter thrombectomy is now the gold standard of acute stroke treatment. Its availability is limited by the lack of trained neuroradiologists in some countries and regions. The role of cardiologists in stroke is expanding from diagnostic help (to reveal the cause of stroke) to acute therapy in those regions, where such up-to-date class IA treatment is not yet available.

## Data Availability

No new data were generated or analysed in support of this research.

## References

[1] Wagner A, Schebesch KM, Isenmann S, Steinbrecher A, Kapapa T, Zeman F, et al Interdisciplinary decision making in hemorrhagic stroke based on CT imaging—differences between neurologists and neurosurgeons regarding estimation of patients’ symptoms, Glasgow Coma Scale, and National Institutes of Health Stroke Scale. Front Neurol 2019;10:997. 10.3389/fneur.2019.00997 31616360 PMC6775244

[2] Manners J, Khandker N, Barron A, Aziz Y, Desai SM, Morrow B, et al An interdisciplinary approach to inhospital stroke improves stroke detection and treatment time. J Neurointerv Surg 2019;11:1080–1084. 10.1136/neurintsurg-2019-014890 31030187

[3] Widimsky P, Doehner W, Diener HC, Van Gelder IC, Halliday A, Mazighi M, et al The role of cardiologists in stroke prevention and treatment: position paper of the European Society of Cardiology Council on Stroke. Eur Heart J 2018;39:1567–1573. 10.1093/eurheartj/ehx478 29020357

[4] Lip GYH, Lane DA, Lenarczyk R, Boriani G, Doehner W, Benjamin LA, et al Integrated care for optimizing the management of stroke and associated heart disease: a position paper of the European Society of Cardiology Council on Stroke. Eur Heart J 2022;43:2442–2460. 10.1093/eurheartj/ehac245 35552401 PMC9259378

[5] Nardai S, Lanzer P, Abelson M, Baumbach A, Doehner W, Hopkins LN, et al Interdisciplinary management of acute ischaemic stroke: current evidence training requirements for endovascular stroke treatment: position paper from the ESC Council on Stroke and the European Association for Percutaneous Cardiovascular Interventions with the support of the European Board of Neurointervention. Eur Heart J 2021;42:298–307. 10.1093/eurheartj/ehaa833 33521827

[6] Nogueira RG, Jadhav AP, Haussen DC, Bonafe A, Budzik RF, Bhuva P, et al Thrombectomy 6 to 24 hours after stroke with a mismatch between deficit and infarct. N Engl J Med 2018;378:11–21. 10.1056/NEJMoa1706442 29129157

[7] Saver JL, Goyal M, van der Lugt A, Menon BK, Majoie CB, Dippel DW, et al Time to treatment with endovascular thrombectomy and outcomes from ischemic stroke: a meta-analysis. JAMA 2016;316:1279–1288. 10.1001/jama.2016.13647 27673305

[8] Cash RE, Boggs KM, Richards CT, Camargo CA Jr, Zachrison KS. Emergency medical service time intervals for patients with suspected stroke in the United States. Stroke 2022;53:e75–e78. 10.1161/STROKEAHA.121.037509 35109679

[9] Choi PMC, Tsoi AH, Pope AL, Leung S, Frost T, Loh PS, et al Door-in-door-out time of 60 minutes for stroke with emergent large vessel occlusion at a primary stroke center. Stroke 2019;50:2829–2834. 10.1161/STROKEAHA.119.025838 31462187

[10] McTaggart RA, Moldovan K, Oliver LA, Dibiasio EL, Baird GL, Hemendinger ML, et al Door-in-door-out time at primary stroke centers may predict outcome for emergent large vessel occlusion patients. Stroke 2018;49:2969–2974. 10.1161/STROKEAHA.118.021936 30571428

[11] Morey JR, Zhang X, Marayati NF, Matsoukas S, Fiano E, Oxley T, et al Mobile interventional stroke teams improve outcomes in the early time window for large vessel occlusion stroke. Stroke 2021;52:e527–e530. 10.1161/STROKEAHA.121.034222 34348472

[12] Widimsky P, Asil T, Abelson M, Koznar B, Tasal A, Roos J, et al Direct catheter-based thrombectomy for acute ischemic stroke: outcomes of consecutive patients treated in interventional cardiology centers in close cooperation with neurologists. J. Am Coll Cardiol 2015;66:487–488. 10.1016/j.jacc.2015.04.076 26205603

[13] Requena M, Ren Z, Ribo M. Direct transfer to angiosuite in acute stroke: why, when, and how? Neurology 2021;97:S34–S41. 10.1212/WNL.0000000000012799 34785602

[14] Requena M, Olivé-Gadea M, Muchada M, Hernández D, Rubiera M, Boned S, et al Direct to angiography suite without stopping for computed tomography imaging for patients with acute stroke: a randomized clinical trial. JAMA Neurol 2021;78:1099–1107. 10.1001/jamaneurol.2021.2385 34338742 PMC8329790

[15] Jovin TG, Nogueira RG, Lansberg MG, Demchuk AM, Martins SO, Mocco J, et al Thrombectomy for anterior circulation stroke beyond 6 h from time last known well (AURORA): a systematic review and individual patient data meta-analysis. Lancet 2022;399:249–258. 10.1016/S0140-6736(21)01341-6 34774198

[16] Nogueira RG, Haussen DC, Liebeskind DS, Jovin TG, Gupta R, Saver JL, et al Clinical effectiveness of endovascular stroke treatment in the early and extended time windows. Int J Stroke 2022;17:389–399. 10.1177/17474930211005740 33705210

[17] Mori E, Yoneda Y, Tabuchi M, Yoshida T, Ohkawa S, Ohsumi Y, et al Intravenous recombinant tissue plasminogen activator in acute carotid artery territory stroke. Neurology 1992;42:976–982. 10.1212/WNL.42.5.976 1579252

[18] The National Institute of Neurological Disorders and Stroke rt-PA Stroke Study Group . Tissue plasminogen activator for acute ischemic stroke. N Engl J Med 1995;333:1581–1587. 7477192 10.1056/NEJM199512143332401

[19] Lees KR, Bluhmki E, von Kummer R, Brott TG, Toni D, Grotta JC, et al Time to treatment with intravenous alteplase and outcome in stroke: an updated pooled analysis of ECASS, ATLANTIS, NINDS, and EPITHET trials. Lancet 2010;375:1695–1703. 10.1016/S0140-6736(10)60491-6 20472172

[20] Ma H, Campbell BCV, Parsons MW, Churilov L, Levi CR, Hsu C, et al Thrombolysis guided by perfusion imaging up to 9 hours after onset of stroke. N Engl J Med 2019;380:1795–1803. 10.1056/NEJMoa1813046 31067369

[21] Lim JC, Churilov L, Bivard A, Ma H, Dowling RJ, Campbell BCV, et al Does intravenous thrombolysis within 4.5 to 9 hours increase clot migration leading to endovascular inaccessibility? Stroke 2021;52:1083–1086. 10.1161/STROKEAHA.120.030661 33588590

[22] Reiff T, Barthel O, Ringleb PA, Pfaff J, Mundiyanapurath S. Safety of mechanical thrombectomy with combined intravenous thrombolysis in stroke treatment 4.5 to 9 hours from symptom onset. J Stroke Cerebrovasc Dis 2020;29:105204. 10.1016/j.jstrokecerebrovasdis.2020.105204 33066886

[23] Furlan A, Higashida R, Wechsler L, Gent M, Rowley H, Kase C, et al Intra-arterial prourokinase for acute ischemic stroke. The PROACT II study: a randomized controlled trial. Prolyse in acute cerebral thromboembolism. JAMA 1999;282:2003–2011. 10.1001/jama.282.21.2003 10591382

[24] Khatri P, Hill MD, Palesch YY, Spilker J, Jauch EC, Carrozzella JA, et al Methodology of the interventional management of stroke III trial. Int J Stroke 2008;3:130–137. 10.1111/j.1747-4949.2008.00151.x 18706007 PMC3057361

[25] Renú A, Millán M, San Román L, Blasco J, Martí-Fŕbregas J, Terceńo M, et al Effect of intra-arterial alteplase vs placebo following successful thrombectomy on functional outcomes in patients with large vessel occlusion acute ischemic stroke. The CHOICE randomized clinical trial. JAMA 2022;327:826–835. 10.1001/jama.2022.1645 35143603 PMC8832304

[26] Zhou Y, Zhang L, Ospel J, Goyal M, McDonough R, Xing P, et al Association of intravenous alteplase, early reperfusion, and clinical outcome in patients with large vessel occlusion stroke: post hoc analysis of the randomized DIRECT-MT trial. Stroke 2022;53:1828–1836. 10.1161/STROKEAHA.121.037061 35240861

[27] LeCouffe NE, Kappelhof M, Treurniet KM, Rinkel LA, Bruggeman AE, Berkhemer OA, et al A randomized trial of intravenous alteplase before endovascular treatment for stroke. N Engl J Med 2021;385:1833–1844. 10.1056/NEJMoa2107727 34758251

[28] Suzuki K, Matsumaru Y, Takeuchi M, Morimoto M, Kanazawa R, et al Effect of mechanical thrombectomy without vs with intravenous thrombolysis on functional outcome among patients with acute ischemic stroke. The SKIP randomized clinical trial. JAMA 2021;325:244–253. 10.1001/jama.2020.23522 33464334 PMC7816103

[29] Campbell BCV, Mitchell PJ, Churilov L, Yassi N, Kleinig TJ, Dowling RJ, et al Tenecteplase versus alteplase before thrombectomy for ischemic stroke. N Engl J Med 2018;378:1573–1582. 10.1056/NEJMoa1716405 29694815

[30] Berkhemer OA, Fransen PSS, Beumer D, van den Berg LA, Lingsma HF, Yoo AJ, et al A randomized trial of intraarterial treatment for acute ischemic stroke. N Engl J Med. 2015;372:11–20. 10.1056/NEJMoa1411587 25517348

[31] Campbell BCV, Mitchell PJ, Kleinig TJ, Dewey HM, Churilov L, Yassi N, et al Endovascular therapy for ischemic stroke with per- fusion-imaging selection. N Engl J Med 2015;372:1009–1018. 10.1056/NEJMoa1414792 25671797

[32] Goyal M, Demchuk AM, Menon BK, Eesa M, Rempel JL, Thorn- ton J, et al Randomized assessment of rapid endovascular treatment of ischemic stroke. N Engl J Med 2015;372:1019–1030. 10.1056/NEJMoa1414905 25671798

[33] Jovin TG, Chamorro A, Cobo E, de Miquel MA, Molina CA, Rovira A, et al Thrombectomy within 8 hours after symptom onset in ischemic stroke. N Engl J Med 2015;372:2296–2306. 10.1056/NEJMoa1503780 25882510

[34] Saver JL, Goyal M, Bonafe A, Diener H-C, Levy EI, Pereira VM, et al Stent-retriever thrombectomy after intravenous t-PA vs. t-PA alone in stroke. N Engl J Med 2015;372:2285–2295. 10.1056/NEJMoa1415061 25882376

[35] Goyal M, Menon BK, van Zwam WH, Dippel DWJ, Mitchell PJ, Demchuk AM, et al Endovascular thrombectomy after large-vessel ischaemic stroke: a meta-analysis of individual patient data from five randomised trials. Lancet 2016;387:1723–1731. 10.1016/S0140-6736(16)00163-X 26898852

[36] Albers GW, Marks MP, Kemp S, Christensen S, Tsai JP, Ortega-Gutierrez S, et al Thrombectomy for stroke at 6 to 16 hours with selection by perfusion imaging. N Engl J Med 2018;378:708–718. 10.1056/NEJMoa1713973 29364767 PMC6590673

[37] Sarraj A, Hassan AE, Savitz S, Sitton C, Grotta J, Chen P, et al Outcomes of endovascular thrombectomy vs medical management alone in patients with large ischemic cores: a secondary analysis of the optimizing patient’s selection for endovascular treatment in acute ischemic stroke (SELECT) study. JAMA Neurol 2019;76:1147–1156. 10.1001/jamaneurol.2019.2109 31355873 PMC6664381

[38] Yoshimura S, Sakai N, Yamagami H, Uchida K, Beppu M, Toyoda K, et al Endovascular therapy for acute stroke with a large ischemic region. N Engl J Med 2022;386:1303–1313. 10.1056/NEJMoa2118191 35138767

[39] Perianen PP, Yan B. Are we ready to offer endovascular thrombectomy to all patients with large ischemic core? Front Neurol 2022;13:893975. 10.3389/fneur.2022.893975 35493819 PMC9043548

[40] Sarraj A, Hassan A, Savitz SI, Grotta JC, Cai C, Parsha KN, et al Endovascular thrombectomy for mild strokes: how low should we go? Stroke 2018;49:2398–2405. 10.1161/STROKEAHA.118.022114 30355094 PMC6209123

[41] Goyal N, Tsivgoulis G, Malhotra K, Ishfaq MF, Pandhi A, Frohler MT, et al Medical management vs mechanical thrombectomy for mild strokes: an international multicenter study and systematic review and meta-analysis. JAMA Neurol 2020;77:16–24. 10.1001/jamaneurol.2019.3112 31545353 PMC6763987

[42] Seners P, Ben Hassen W, Lapergue B, Arquizan C, Heldner MR, Henon H, et al Prediction of early neurological deterioration in individuals with Minor stroke and large vessel occlusion intended for intravenous thrombolysis alone. JAMA Neurol 2021;78:321–328. 10.1001/jamaneurol.2020.4557 33427887 PMC7802007

[43] Zaidat OO, Mueller-Kronast NH, Hassan AE, Haussen DC, Jadhav AP, Froehler MT, et al Impact of balloon guide catheter use on clinical and angiographic outcomes in the STRATIS stroke thrombectomy registry. Stroke 2019;50:697–704. 10.1161/STROKEAHA.118.021126 30776994

[44] Brinjikji W, Starke RM, Murad MH, Fiorella D, Pereira VM, Goyal M, et al Impact of balloon guide catheter on technical and clinical outcomes: a systematic review and meta-analysis. J Neurointerv Surg 2018;10:335–339. 10.1136/neurintsurg-2017-013179 28754806

[45] Lapergue B, Blanc R, Gory B, Labreuche J, Duhamel A, Marnat G, et al Effect of endovascular contact aspiration vs stent retriever on revascularization in patients with acute ischemic stroke and large vessel occlusion: the aster randomized clinical trial. JAMA 2017;318:443–452. 10.1001/jama.2017.9644 28763550 PMC5817613

[46] Nogueira RG, Frei D, Kirmani JF, Zaidat O, Lopes D, Turk AS III, et al Safety and efficacy of a:3. -dimensional stent retriever with aspiration-based thrombectomy vs aspiration-based thrombectomy alone in acute ischemic stroke intervention: a randomized clinical trial. JAMA Neurol 2018;75:304–311. 10.1001/jamaneurol.2017.3967 29296999 PMC5885851

[47] Turk AS III, Siddiqui A, Fifi JT, De Leacy RA, Fiorella DJ, Gu E, , et al Aspiration thrombectomy versus stent retriever thrombectomy as first-line approach for large vessel occlusion (compass): a multicentre, randomised, open label, blinded outcome, non-inferiority trial. Lancet 2019;393:998–1008. 10.1016/S0140-6736(19)30297-1 30860055

[48] Mohammaden MH, Haussen DC, Perry da Camara C, Pisani L, Olive Gadea M, Al-Bayati AR, et al Hyperdense vessel sign as a potential guide for the choice of stent retriever versus contact aspiration as first-line thrombectomy strategy. J Neurointerv Surg 2021;13:599–604. 10.1136/neurintsurg-2020-016005 32737205

[49] Bourcier R, Mazighi M, Labreuche J, Fahed R, Blanc R, Gory B, et al Susceptibility vessel sign in the aster trial: higher recanalization rate and more favourable clinical out- come after first line stent retriever compared to contact aspiration. J Stroke 2018;20:268–276. 10.5853/jos.2018.00192 29886714 PMC6007297

[50] Langezaal LCM, van der Hoeven EJRJ, Mont’Alverne FJA, de Carvalho JJF, Lima FO, Dippel DWJ, et al Endovascular therapy for stroke due to basilar-artery occlusion. N Engl J Med 2021;384:1910–1920. 10.1056/NEJMoa2030297 34010530

[51] Liu X, Dai Q, Ye R, Zi W, Liu Y, Wang H, et al Endovascular treatment versus standard medical treatment for vertebrobasilar artery occlusion (BEST): an open-label, randomised controlled trial. Lancet Neurol 2020;19:115–122. 10.1016/S1474-4422(19)30395-3 31831388

[52] Tao C, Nogueira RG, Zhu Y, Sun J, Han H, Yuan G, et al Trial of endovascular treatment of acute basilar-artery occlusion. N Engl J Med 2022; 387:1361–1372. 10.1056/NEJMoa2206317 36239644

[53] Da Ros V, Scaggiante J, Pitocchi F, Sallustio F, Lattanzi S, Umana GE, et al Mechanical thrombectomy in acute ischemic stroke with tandem occlusions: impact of extracranial carotid lesion etiology on endovascular management and outcome. Neurosurg Focus 2021;51:E6. 10.3171/2021.4.FOCUS21111 34198245

[54] Haussen DC, Turjman F, Piotin M, Labreuche J, Steglich-Arnholm H, Holtmannspötter M, et al Head or neck first? Speed and rates of reperfusion in thrombectomy for tandem large vessel occlusion strokes. Interv Neurol 2020; 8:92–100. 10.1159/000496292 32508890 PMC7253855

[55] Zinkstok SM, Roos YB. Early administration of aspirin in patients treated with alteplase for acute ischaemic stroke: a randomised controlled trial. Lancet 2012;380:731–737. 10.1016/S0140-6736(12)60949-0 22748820

[56] Papanagiotou P, Haussen DC, Turjman F, Labreuche J, Piotin M, Kastrup A, et al Carotid stenting with antithrombotic agents and intracranial thrombectomy leads to the highest recanalization rate in patients with acute stroke with tandem lesions. JACC Cardiovasc Interv 2018;11:1290–1299. 10.1016/j.jcin.2018.05.036 29976365

[57] Gory B, Haussen DC, Piotin M, Steglich-Arnholm H, Holtmannspötter M, Labreuche J, et al Impact of intravenous thrombolysis and emergent carotid stenting on reperfusion and clinical outcomes in patients with acute stroke with tandem lesion treated with thrombectomy: a collaborative pooled analysis. Eur J Neurol 2018;25:1115–1120. 10.1111/ene.13633 29575634

[58] Sadeh-Gonik U, Tau N, Friehmann T, Bracard S, Anxionnat R, Derelle AL, et al Thrombectomy outcomes for acute stroke patients with anterior circulation tandem lesions: a clinical registry and an update of a systematic review with meta-analysis. Eur J Neurol 2018;25:693–700. 10.1111/ene.13577 29350803

[59] Stampfl S, Ringleb PA, Möhlenbruch M, Hametner C, Herweh C, Pham M, et al Emergency cervical internal carotid artery stenting in combination with intracranial thrombectomy in acute stroke. Am J Neuroradiol 2014;35:741–746. 10.3174/ajnr.A3763 24157733 PMC7965815

[60] Menon BK, Hill MD, Davalos A, Roos YBWEM, Campbell BCV, Dippel DWJ, et al Efficacy of endovascular thrombectomy in patients with M2 segment middle cerebral artery occlusions: meta-analysis of data from the HERMES collaboration. J Neurointerv Surg 2019;11:1065–1069. 10.1136/neurintsurg-2018-014678 30975736

[61] Grossberg JA, Rebello LC, Haussen DC, Bouslama M, Bowen M, Barreira CM, et al Beyond large vessel occlusion strokes: distal occlusion thrombectomy. Stroke 2018;49:1662–1668. 10.1161/STROKEAHA.118.020567 29915125

[62] Sandercock PA, Counsell C, Kane EJ. Anticoagulants for acute ischaemic stroke. Cochrane Database Syst Rev. 2015;2015(3):CD000024. 10.1002/14651858.CD000024.pub4 25764172 PMC7065522

[63] Yang M, Huo X, Gao F, Wang A, Ma N, Liebeskind DS, et al Safety and efficacy of heparinization during mechanical thrombectomy in acute ischemic stroke. Front Neurol 2019;10:299. 10.3389/fneur.2019.00299 30984103 PMC6450216

[64] Chalos V, van de Graaf RA, Roozenbeek B, van Es ACGM, den Hertog HM, Staals J, et al Multicenter randomized clinical trial of endovascular treatment for acute ischemic stroke. The effect of periprocedural medication: acetylsalicylic acid, unfractionated heparin, both, or neither (MR CLEAN-MED). Rationale and study design. Trials 2020;21:644. 10.1186/s13063-020-04514-9 32665035 PMC7362523

[65] Simonsen CZ, Yoo AJ, Sørensen LH, Juul N, Johnsen SP, Andersen G, et al Effect of general anesthesia and conscious sedation during endovascular therapy on infarct growth and clinical outcomes in acute ischemic stroke. JAMA Neurol 2018;75:470–477. 10.1001/jamaneurol.2017.4474 29340574 PMC5885172

[66] Cappellari M, Pracucci G, Forlivesi S, Saia V, Nappini S, Nencini P, et al General anesthesia versus conscious sedation and local anesthesia during thrombectomy for acute ischemic stroke. Stroke 2020;51:2036–2044. 10.1161/STROKEAHA.120.028963 32517584

[67] Matusevicius M, Cooray C, Bottai M, Mazya M, Tsivgoulis G, Nunes AP, et al Blood pressure after endovascular thrombectomy: modelling for outcomes based on recanalization status. Stroke 2020;51:519–525. 10.1161/STROKEAHA.119.026914 31822252

[68] Bath PM, Song L, Silva GS, Mistry E, Petersen N, Tsivgoulis G, et al Blood pressure management for ischemic stroke in the first 24 hours. Stroke 2022;53:1074–1084. 10.1161/STROKEAHA.121.036143 35291822 PMC11288209

[69] Sun D, Huo X, Raynald JB, Tong X, Ma G, et al Predictors of symptomatic intracranial hemorrhage after endovascular treatment for acute large vessel occlusion: data from ANGEL-ACT registry. J Thromb Thrombolysis 2022;54:558–565. 10.1007/s11239-022-02688-4 35913684

